# Role of Crushable Biochar in the Micro and Macro Mechanical Behaviour of Biochar-Amended Soil: A DEM Study

**DOI:** 10.3390/ma18204700

**Published:** 2025-10-14

**Authors:** Yuanbing Xia, Zhilin Ren, Gang Wei, Yingkang Yao

**Affiliations:** 1 State Key Laboratory of Precision Blasting, Jianghan University, Wuhan 430056, China; 221604010079@hhu.edu.cn (Y.X.); shanxiyao@jhun.edu.cn (Y.Y.); 2 Hubei Key Laboratory of Blasting Engineering, Jianghan University, Wuhan 430056, China; 3 Key Laboratory of Ministry of Education for Geomechanics and Embankment Engineering, Hohai University, Nanjing 210098, China; 4 Key Laboratory of Environmental Geology of Qinghai Province, Qinghai Bureau of Environmental Geology Exploration, Xining 810007, China; harley0701@163.com; 5 Qinghai 906 Engineering Survey and Design Institute Co., Ltd., Xining 810007, China

**Keywords:** biochar, compressive properties, particle fragmentation, MatDEM, numerical simulation

## Abstract

This study investigates the microscale mechanisms underlying the compressibility of biochar-amended soils through combined discrete element method (DEM) simulations and laboratory consolidation tests. A three-dimensional discrete element model was established based on the MatDEM platform, accounting for the particle crushing process of biochar particles and its impact on soil mechanical properties. The biochar agglomerate particles generated in the simulation exhibit irregular morphology, and particles within different size ranges were selected for investigation. According to the model and experimental results, the average relative error is about 7%. Results demonstrate that moderate biochar content effectively reduces soil compressibility by enhancing load transfer through stable force chains formed by biochar particles, which exhibit larger contact areas and higher stiffness compared to native soil particles. However, when the biochar content exceeds approximately 40%, particle crushing intensifies, particularly under high initial void ratios, leading to increased soil compressibility. Furthermore, a larger initial void ratio weakens interparticle confinement, promotes microcrack propagation, and thereby exacerbates compressive deformation. Biochar fragmentation progresses through three stress-dependent stages: initial compaction (<100 kPa), skeletal damage (100–800 kPa), and crushing saturation (>800 kPa). Increased biochar particle size correlates with higher fragmentation rates, refined particle gradation, and reduced coordination numbers, collectively weakening the soil skeleton and promoting deformation. These findings underscore the importance of optimizing biochar content and applying graded loading strategies to balance enhanced soil performance with material integrity. These findings emphasize the necessity of optimizing biochar application rates to balance enhanced soil performance with resource efficiency, providing critical insights for sustainable geotechnical practices.

## 1. Introduction

The increasing concern on resource conservation and sustainable development has intensified interest in strategies for waste soil improvement and remediation. Among emerging approaches, biochar, a porous, lightweight material characterized by a high specific surface area, strong adsorption capacity, and exceptional water retention, has gained attention for its multifunctional role in soil enhancement, contaminant remediation, and carbon sequestration [1,2,3,4,5,6,7,8]. Owing to its capacity to address multiple environmental and engineering challenges, biochar has become a promising component in sustainable geotechnical practice.

Many studies have examined the effects of biochar on key soil engineering properties, such as compressibility, permeability, shear strength, and cohesion, while systematically assessing variables including admixture ratio and particle size [9,10,11,12]. However, most of these investigations rely on conventional geotechnical testing methods that primarily capture macroscopic responses. Such approaches offer limited resolution to reveal microscale structural changes in soil particles and pore networks, thereby constraining mechanistic understanding of deformation processes, especially under complex loading conditions.

Although advanced imaging techniques, such as X-ray computed tomography (CT), have enabled limited insights into microscale deformation [13,14], their high cost and technical complexity hinder widespread application. In contrast, the discrete element method (DEM) provides a computationally efficient and versatile framework for simulating granular media, offering explicit modeling of particle-scale interactions, discontinuities, and large deformations inherent to geomaterials [15,16]. DEM’s capacity to define particle density, Young’s modulus, and intergranular friction [17] makes it particularly suitable for analyzing biochar amend soil composites. Particularly, the brittle nature of biochar induces particle crushing during compression often overlooked in traditional laboratory testing.

Recent developments in DEM have demonstrated its effectiveness in capturing micro–macro mechanical relationships in geomechanics. Applications to triaxial tests (Sensitivity Analysis of Fine-Scale Parameters, 2024), uniaxial compaction (A Local Constitutive Model for DEM, 2015), and critical state behavior (Imperial College London, 2024) have validated its predictive capability. By reconstructing soil architectures through particle aggregation and cementation models [18,19,20,21,22,23], DEM enables real-time monitoring of force chains, contact stresses, and pore evolution, capabilities unattainable through physical experiments alone. Nevertheless, DEM investigations into the compressibility of biochar-amended soils remain scarce, particularly under high void ratio conditions where biochar is prone to brittle fracture. The implications of such fracture behavior for the overall mechanical response of the soil remain insufficiently understood.

To address this gap, this study integrates DEM simulations with laboratory consolidation tests to elucidate the microscale mechanisms governing the compressibility of high void ratio biochar–soil mixtures. A three-dimensional DEM framework was implemented in the MatDEM platform to examine the influence of biochar content, initial void ratio, and particle crushing on macroscopic compressibility, force transmission, and pore structure evolution. By correlating microstructural processes, including particle rearrangement and biochar fragmentation with mechanical behavior, this work proposes optimized biochar characteristics and application strategies for sustainable soil stabilization.

## 2. Validation of Discrete Element Simulations

### 2.1. Mechanism Governing the Behaviour of Biochar-Amended Soils

Biochar exhibits unique physicochemical properties, including low density, high porosity, large specific surface area, alkaline pH, high cation exchange capacity (CEC), and remarkable structural stability. When incorporated into soil, these attributes collectively modify the physical and mechanical behavior of the soil matrix. The low bulk density and abundant meso- to macropores of biochar reduce overall soil density while improving pore structure and distribution. At the same time, the rigid and porous skeleton of biochar particles enables more efficient stress transfer, thereby reducing compressibility and accelerating pore water pressure dissipation during consolidation. In terms of strength, the rough and angular surfaces of biochar enhance interparticle friction, while its interactions with clay minerals and organic matter promote aggregate formation, resulting in increased shear strength and structural stability. Chemically, the alkaline nature and high CEC of biochar buffer soil acidity, improve nutrient retention, and indirectly reinforce aggregate stability and long-term resistance to disturbance. These effects, however, are dose-dependent: moderate incorporation generally strengthens soil structure and stability, whereas excessive application may induce hydrophobicity or particle fragmentation, ultimately diminishing mechanical performance. Overall, the mechanisms by which biochar regulates soil behavior arise from integrated modifications to pore structure, stress-transfer pathways, and chemical conditions, producing coupled effects on density, compressibility, water retention, and strength.

### 2.2. Principles of MatDEM

The DEM, initially introduced by Cundall in 1971 for rock mechanics analysis and later extended to soils by Cundall and Strack in 1979, proves effective in modeling discontinuities, inhomogeneities, and large deformation damages within rock and soil bodies [24,25]. MatDEM 4.51 employs a matrix-based discrete element algorithm and a three-dimensional contact algorithm, enabling approximately 15 million three-dimensional element motion calculations per second (up to 40 million for two-dimensional calculations). Its computational unit scale and processing speed surpass other mainstream commercial software by over 30 times (capable of handling 3 million 3D units or 10 million 2D units), achieving a breakthrough in efficient discrete element numerical simulation of millions of particles [26]. The software features automatic stacking modeling, layered material assignment, nodal surface and load setting, and offers robust post-processing functions and secondary development capabilities. Its rapid calculation speed, large number of calculation units, and accurate simulation of granular unit behavior under various conditions render it widely applicable in mining, civil engineering, and water conservancy sectors [27,28,29].

**Figure 1:**
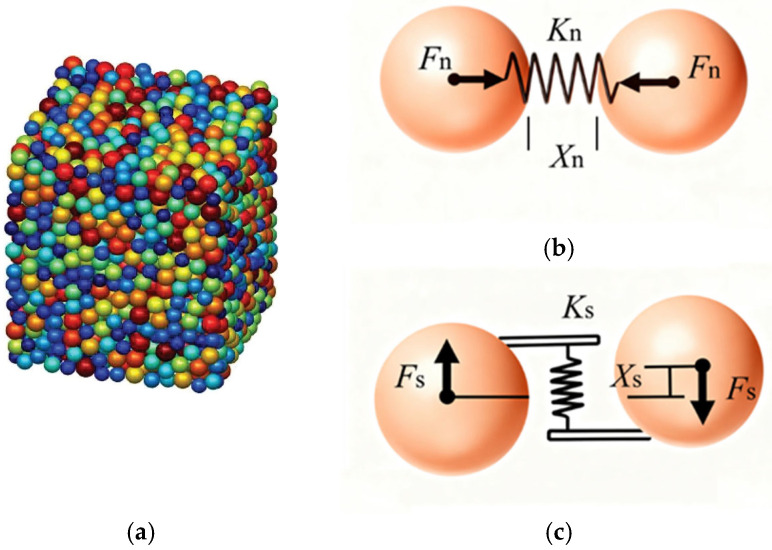
Schematic diagram of the linear elastic model: (**a**) A 3D stacking model; (**b**) normal contact; (**c**) tangential contact.

Figure 1 illustrates a schematic diagram of the linear elastic model. As depicted in Figure 1a, the DEM constructs a geotechnical model by aggregating and binding a series of particles with specific mechanical properties. Subsequently, numerical simulation is conducted through a time-step iterative algorithm to simulate the deformation and damage processes of macroscopic rocks and soils [21]. The linear elastic contact model represents the most fundamental and widely utilized model. Illustrated in Figure 1b, this model assumes that cells interact with each other via spring forces. The normal force (*F*_n_) and normal deformation (*X*_n_) between two cells can be simulated by a normal spring [30,31], calculated as:

(1) $$F_{n}=\left\{\begin{array}{c} K_{n}X_{n},\;\left(X_{n}{<X}_{b}\right)\;intact\\ K_{n}X_{n},\;\left(X_{n}<0\right)\;broken\\ 0\;\left(X_{n}>0\right)\;broken \end{array}\right.$$

(2) $$K_{n}=\frac{\surd 2Ed}{4(1-2\gamma )}$$

(3) $$X_{n}=X_{n}^{f}+∆X_{n}$$

(4) $$∆X_{n}=∆t\cdot V_{n}+\frac{1}{2}∆t^{2}\cdot a_{n}$$

where $K_{n}$ is normal stiffness, $X_{n}$ is relative normal displacement, $X_{b}$ is breaking displacement, *E* shows the elasticity modulus, *d* indicates the particle diameter, *ν* stands for Poisson’s ratio, $X_{n}^{f}$ refers to the former relative normal displacement, $∆X_{n}$ denotes the incremental relative normal displacement, Δ*t* shows the time step, $V_{n}$ represents the normal velocity, and $a_{n}$ stands for the normal acceleration.

When the relative normal displacement, $X_{n}$, exceeds the breaking one, $X_{b}$, the connection between the particles breaks up and the tension disappears. Thus the maximum normal force between the particles is expressed as:

(5) $$F_{nmax}=K_{n}X_{b}$$

(6) $$X_{b}=\frac{{3K}_{n}+K_{s}}{6\surd 2K_{n}(K_{n}+K_{s})}T_{u}\cdot d^{2}$$

(7) $$K_{s}=\frac{\surd 2(1-5v)Ed}{4(1+v)(1-2v)}$$

where $T_{u}$ is tensile strength, $K_{s}$ is shear stiffness.

Similarly, the shear force ($F_{s}$) and the shear relative displacement ($X_{s}$) between the two particles are simulated by the shear springs [32], calculated by:

(8) $$F_{s}=K_{s}X_{s}$$

(9) $$F_{smax}=F_{s0}-\mu_{p}F_{n}$$

where $F_{smax}$ is the maximum shear force; $F_{s0}$ is the initial inter-element shear resistance; and $\mu_{p}$ is the inter-element coefficient of friction. The intact shear connection between both particles also breaks once the external force exceeds $F_{smax}$.

**Figure 2:**
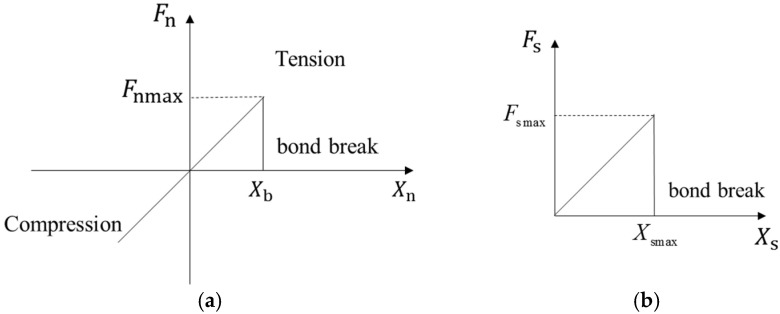
(**a**) Normal force and normal displacement; (**b**) shear force and shear displacement.

Figure 2 depicts the specific relationship between normal force and normal displacement, as well as shear force and shear displacement between the elements.

### 2.3. Determination of Parameters

Table 1 presents the physical and mechanical parameters of the soil and biochar used in this study. The biochar was produced from bamboo through a biomass pyrolysis process, while the soil sample was clay with a particle size range of 0~0.005 mm. The initial parameter values applied in the simulations were derived from experimental measurements and supplemented by relevant literature. Since macroscopic mechanical parameters often fall below the default values defined in particle-stacking models, material training is required to obtain more accurate mechanical properties. Liu et al. (2017) [33] proposed conversion formulas linking macro- and micro-scale mechanical parameters. By applying these formulas together with the automated training module, MatDEM software (version 3.0, developed by Professor Chun Liu’s team at Nanjing University, Nanjing, China) can automatically generate the specific mechanical properties of geomaterials. The core principle lies in continuously adjusting micro-scale parameters to reproduce the desired macroscopic behavior of the sample. The analytical solution for the relationship between macro- and micro-scale parameters is as follows:

(10) $$F_{s0}=\frac{1-\surd 2\mu_{p}}{6}C_{u}\cdot d^{2}$$

(11) $$\mu_{p}=\frac{-2\sqrt{2}+\surd 2{\left[\sqrt{\left(1+\mu_{i}^{2}\right)}+\mu_{i}\right]}^{2}}{2+2{\left[\sqrt{\left(1+\mu_{i}^{2}\right)+\mu_{i}}\right]}^{2}}$$

where elastic modulus (*E*), Poisson’s ratio (*ν*), uniaxial tensile strength ($T_{u}$), uniaxial compressive strength ($C_{u}$), and internal friction coefficient ($\mu_{i}$) are the macroscopic mechanical parameters of the material.

### 2.4. Numerical Modeling

**Figure 3:**
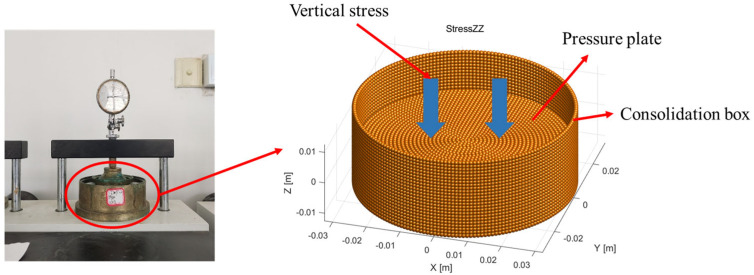
Numerical model of consolidation test for mixed soil samples.

Figure 3 shows the numerical model used for the consolidation test of mixed soil samples. To achieve more accurate numerical simulation, a three-dimensional discrete element model was developed based on the indoor soil consolidation test. The model comprises a consolidation box and the soil sample. The consolidation box includes a side-confining tube and upper and lower pressure plates. The model has a diameter of 61.8 mm and a height of 20 mm, containing a total of 162,524 particles. Biochar particles were modeled as aggregates and uniformly mixed with soil particles. The side-confining tube and lower pressure plate were fixed in place. To replicate the loading procedure of the laboratory test, pressure was applied incrementally through the upper pressure plate, mimicking the addition of weights on top of the consolidation box. The loading process was divided into 12 stages, with stress fully equilibrated through iterative calculations after each stage, and the results recorded accordingly.

The initial void ratio was also considered to account for the effects of high void ratio on both soil and biochar. The influence of void ratio was indirectly reflected in the simulation by adjusting key parameters, such as contact force, friction, and bonding forces between particles. These adjustments influenced the inter-particle contact forces and overall mechanical behavior. When the biochar content was set to zero, this condition was achieved by controlling the particle generation density and packing method, that is, by placing fewer soil particles within the sample space to create a very loose packing state. The model was then validated against existing experimental results, and its accuracy and reliability were ensured through iterative adjustments.

### 2.5. Simulated Crushing of Biochar Pellets

The macroscopic mechanical behavior of granular materials, including compressibility and stress–strain response, is strongly influenced by the extent of particle crushing during loading [36,37,38,39]. This effect, in turn, depends on factors such as particle shape, microstructure, and size [40,41]. For biochar-amended soils, compressibility is also closely linked to the intrinsic properties of the biochar, which vary with feedstock type and pyrolysis conditions. In this study, the biochar was derived from bamboo and produced under controlled pyrolysis conditions. Although this biochar differs from that used in other studies (e.g., Reddy et al., 2015) [9], the primary focus here is on the general trend of strain behavior with varying biochar content. Future work may further explore its influence on absolute strain values.

The DEM plays a crucial role in studying the impact of particle fragmentation on soil mechanical properties from a microscopic perspective [42,43,44,45], as it accounts for various particle interactions, including friction, cohesion, and damage, while providing particle-scale force and velocity data [42,43,44,45,46]. Additionally, some researchers have used DEM simulations to show that bond strength between particles and changes in particle size distribution due to fragmentation can alter the critical state of the soil, thereby affecting its compressibility and shear strength [47,48].

**Figure 4:**
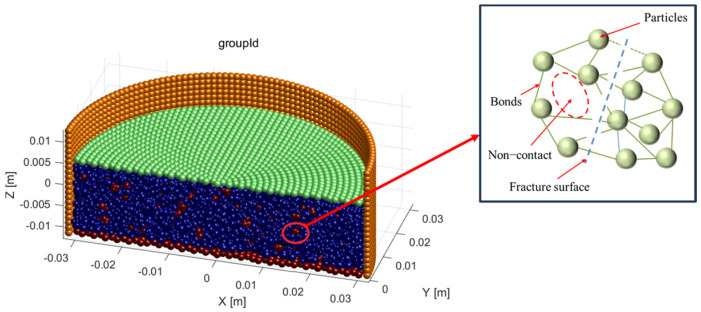
Fragmentation of biochar particles (showing joining and breaking of particles by decreasing the radius). Blue represents soil particles, and red represents biochar particles.

**Figure 5:**
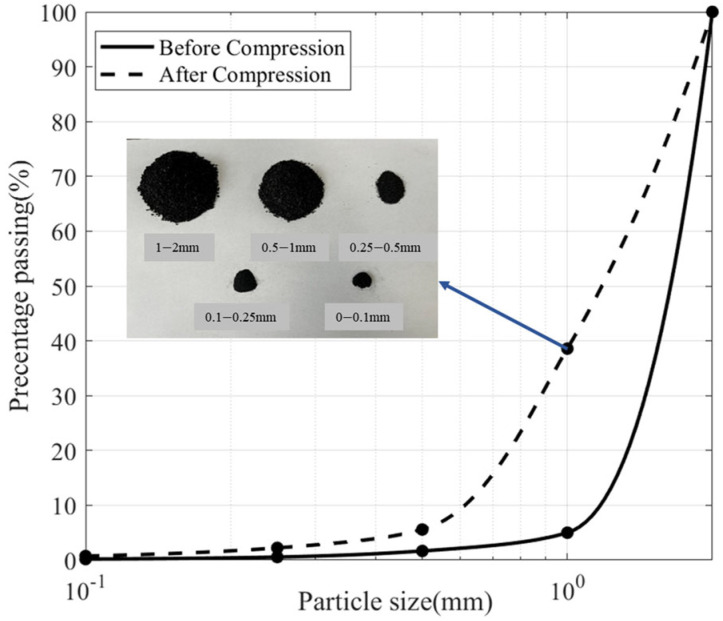
Particle size distribution of 1−2 mm biochar after consolidation.

Figure 4 illustrates the fragmentation process of biochar particles. In this study, smaller spherical particles were bonded into agglomerates to simulate the crushing behavior of biochar by breaking the bonds between the individual particles. Figure 5 demonstrates the crushing of 1~2 mm biochar particles after consolidation. During side-limit compression tests on biochar-amended soil samples, biochar particles may be crushed under pressure, especially as the biochar content increases. This crushing effect becomes more severe with higher applied loads. As a result, the proportion of fine particles increases, leading to changes in the particle size distribution of the mixed soil. Ng et al. (2022) [49] reported from their study on the mechanical properties of biochar-amended completely decomposed granite that the crushing of biochar particles contributes to a reduction in the peak stress of biochar–soil mixtures. This finding suggests that the crushing effect of biochar should be taken into account when evaluating the compressive behavior of such soils.

## 3. Results

### 3.1. Comparison of Experimental and Numerical Simulation

**Figure 6:**
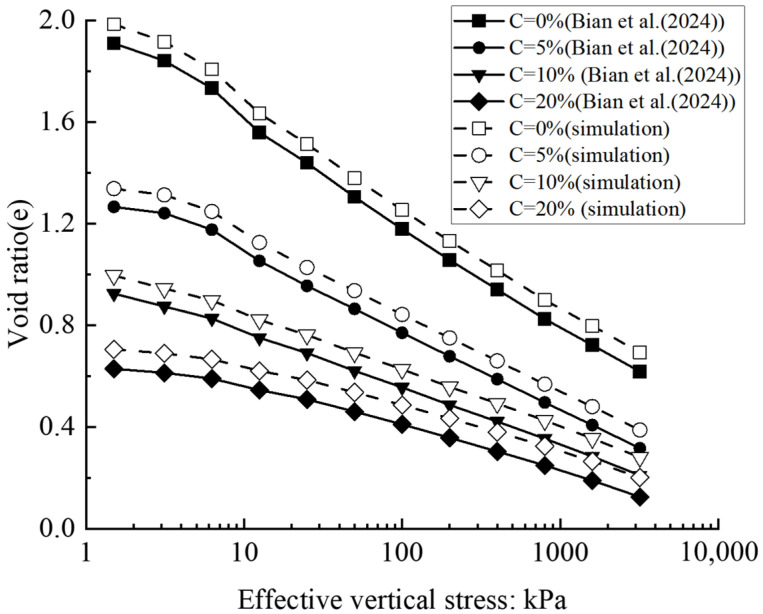
Comparison of numerical simulation and experimental compression curves with different influencing factors [7].

Bian et al. (2024) [7] applied biochar to the treatment of soil and investigated the effects of biochar content, particle size, and initial void ratio on the physical properties of the soil. Their findings provided valuable references for the numerical simulations conducted in this study. Figure 6 depicts the comparison between the compression curves obtained from the numerical simulation and the experiment. In soil without biochar, the initial structure is typically quite loose, exhibiting limited skeletal stability, which leads to continuous deformation accumulation. Additionally, ongoing water drainage from the soil further contributes to the increased deformation. In the simulation, soil and biochar particles were uniformly mixed. Aggregates of varying sizes were generated, and a pressure plate was applied in a stepwise loading procedure, closely replicating the laboratory testing conditions.

It is worth noting that the porosity obtained from numerical simulations was slightly higher than experimental results in certain cases. The average relative error was approximately 7%, calculated using the formula shown below. This discrepancy may partly stem from the simulation not fully accounting for the water absorption and expansion behavior of biochar. In the experiments, the micro-expansion of biochar upon water uptake contributes to increased mixture density; however, this effect is not explicitly represented in the simulation, resulting in a modest overestimation of porosity. Nevertheless, the model demonstrates strong agreement with experimental trends overall, thereby validating its rationality and reliability.

(12) $$MRPE=\frac{1}{n}\sum \frac{S_{i}-E_{i}}{E_{i}}\times 100\%$$

with the symbols representing the following factors: Mean Relative Percentage Error (MRPE), number of data points (n), simulated value at the i-th data point ($S_{i}$), experimental value at the i-th data point ($E_{i}$).

### 3.2. Parametric Analysis

#### 3.2.1. Biochar Content

**Figure 7:**
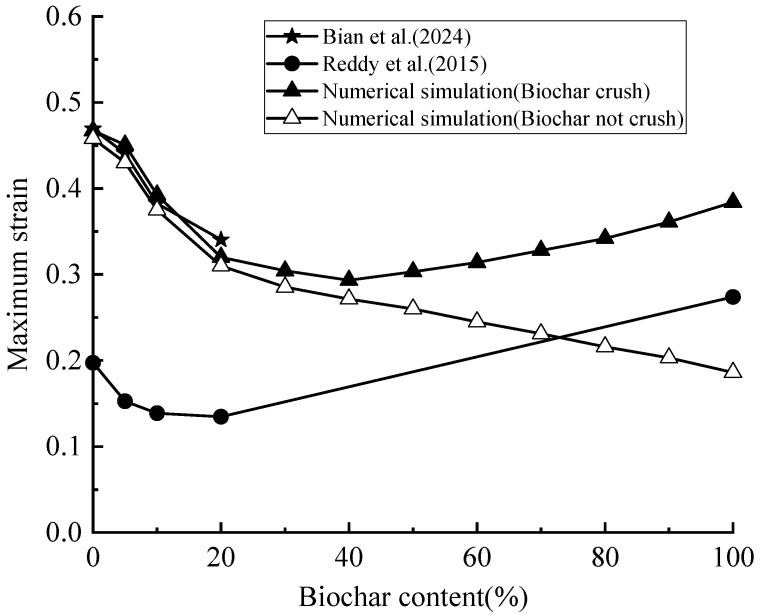
Final strain values of mixed soil samples with different biochar content [7,9].

Figure 7 illustrates the effect of biochar content on soil compressibility. At a biochar content of 40%, the ultimate strain of the biochar–soil mixture decreased by 39.8% relative to untreated soil. In contrast, at 100% biochar content, the ultimate strain increased by 30.9% compared with the 40% treatment. Numerical simulations indicate that the incorporation of an appropriate amount of biochar effectively reduces soil compressibility, consistent with previous studies on biochar-amended soils [50,51]. It should be noted that the numerical simulations and the experiments by Bian et al. (2024) [7] were conducted using standard oedometer rings (diameter 61.8 mm, height 20 mm), whereas Reddy et al. (2015) [9] employed a larger ring (diameter 70 mm, height 140 mm). Consequently, the maximum strain values reported by Reddy et al. (2015) [9] are not directly comparable with the simulation results; however, the overall trend of strain versus biochar content remains informative.

By integrating numerical and experimental results, a nonlinear effect of biochar on soil compressibility is observed: soil strain initially decreases with increasing biochar content, but when the content exceeds a critical threshold, the maximum strain increases. This behavior is attributed to the fact that biochar initially enhances soil structure, whereas excessive amounts may induce adverse effects such as particle breakage, thereby reducing its ameliorative effect [52]. The underlying mechanisms of this nonlinear response are discussed in detail in subsequent sections.

#### 3.2.2. Biochar Particle Size

**Figure 8:**
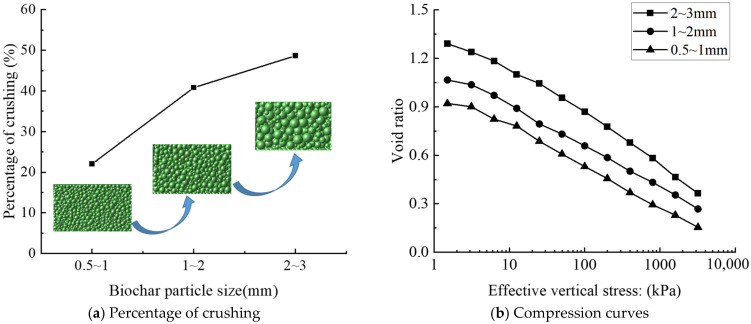
Particle fragmentation rate of mixed soils with different biochar particle sizes.

Figure 8 illustrates the relationship between soil compressibility and particle fragmentation rate for different biochar particle sizes. The results indicate that the fragmentation rate of biochar increases with particle size, which in turn elevates the compressibility of the biochar–soil mixture. This trend can be attributed to two primary mechanisms. First, larger particles possess greater surface area and exhibit more uneven internal stress distribution under external loading, rendering them more susceptible to breakage. Second, larger biochar particles introduce larger pores into the soil matrix, thereby increasing the overall compressibility of the mixture. Similar observations have been reported in previous studies [53,54,55].

**Figure 9:**
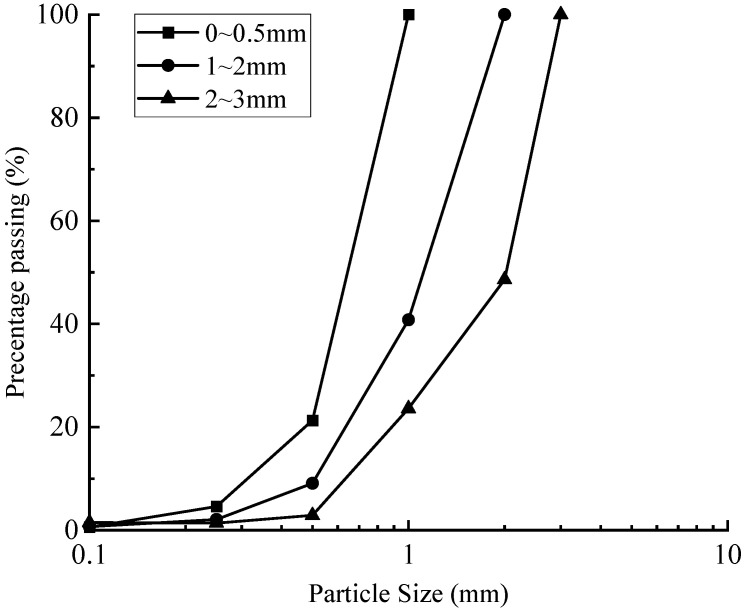
Distribution of different particle sizes of biochar after consolidation.

Figure 9 depicts the post-consolidation particle size distribution of biochar, showing that the proportion of particles undergoing crushing increases with initial particle size, whereas the proportion of fine particles produced from crushing decreases progressively. Larger particles predominantly fragment into the next smaller size class along with a portion of finer particles. The improved gradation resulting from this process reduces subsequent fragmentation, as particle size strongly influences re-crushing potential. Smaller particles are less likely to undergo further breakage, whereas under identical compressive stress, larger particles exhibit a higher re-crushing rate than smaller ones [56,57,58].

#### 3.2.3. Initial Void Ratio

**Figure 10:**
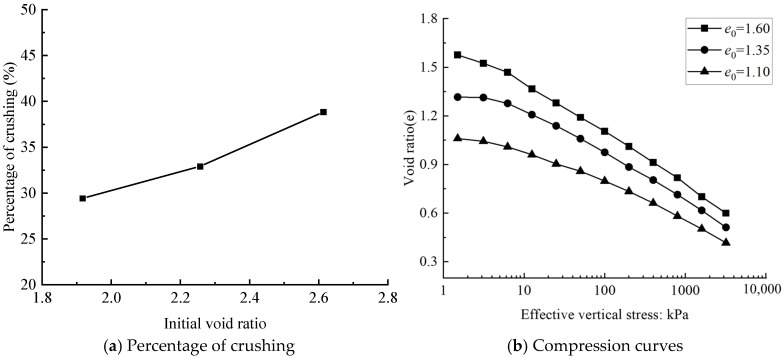
Relationship between compressibility and particle fragmentation rate of mixed soils with different initial void ratio.

Figure 10 shows the relationship between soil compressibility and particle fragmentation rate in biochar–soil mixtures with varying initial void ratios (*e*_0_). At a consolidation pressure of 400 kPa, an increase in the initial void ratio corresponds to a higher post-consolidation fragmentation rate of biochar particles, which in turn increases the compressibility of the mixture. This behavior can be explained by two primary mechanisms. First, a higher void ratio reflects a looser soil structure, enabling the porous biochar particles to form more effective drainage channels and thereby facilitating water dissipation during compression [54]. Second, a larger void ratio reduces the number of particle–particle contact points, weakening interparticle constraint forces. This reduction in confinement increases particle mobility, allowing greater slippage and rearrangement under load. Consequently, biochar particles experience higher breakage rates, further amplifying the compressibility of the mixture.

#### 3.2.4. Average Coordination Number

**Figure 11:**
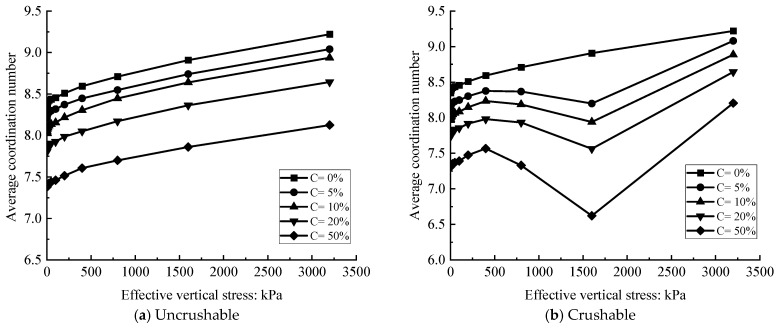
Variation in average coordination number with different biochar content.

Particle fragmentation is strongly influenced by the coordination number between particles [59]. Figure 11 presents the variation in average coordination number for biochar–soil mixtures with different biochar contents. At the initial loading stage, the average coordination number increases with pressure, indicating enhanced particle contacts. However, beyond a pressure of 400 kPa, the coordination number declines, primarily due to substantial particle breakage, which disrupts existing contacts. Similar findings were reported by Minh et al. (2013) [60] through discrete element simulations, which showed that particle breakage alters the particle-size distribution, initially increasing coordination number but subsequently reducing the likelihood of further breakage during later compression stages. Once biochar fragmentation ceases and compaction progresses, the coordination number increases again as particles reorganize into denser arrangements.

For intact particles, continuous compaction maintains a positive correlation between coordination number and applied pressure. Notably, higher biochar content results in a lower average coordination number, a trend attributed to the pronounced size disparity between biochar and soil particles. As biochar proportion increases, sample porosity rises, reducing the number of effective interparticle contacts.

## 4. Discussions

### 4.1. The Effect of Internal Force Evolution on the Compressive Properties of Soils

The force chain serves as a critical link between the macroscopic and microscopic properties of granular materials, providing a foundation for multi-scale mechanical investigations of granular systems [61,62,63]. In MatDEM, the contact interactions between particles are characterized through the neighbor matrix and attribute matrix, which record the magnitude and direction of the resultant forces at particle contact points. These datasets enable the construction of force chain diagrams, offering a direct visual representation of force transmission within the soil matrix. Typically, contact forces concentrate on particles with larger diameters due to their greater contact area relative to smaller particles. As a result, larger particles more readily establish stable force chain networks, leading to preferential stress concentration on these particles [64,65,66,67,68].

**Figure 12:**
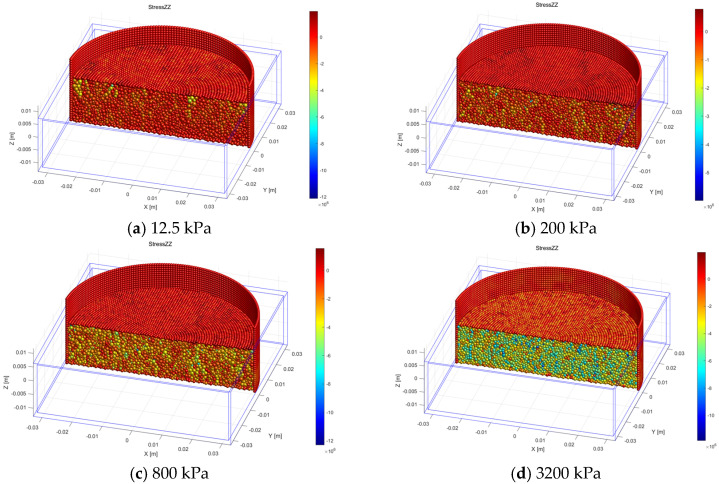
Internal stress distribution diagram of soil specimen under partial loading (section).

Figure 12 shows the vertical stress distribution within particles under partial loading, with representative critical loading stages selected for clarity. At low pressures (e.g., 12.5 kPa), stresses are minimal and uniformly distributed, as indicated by lighter colors, reflecting an initial contact state without pronounced force chain formation. As loading increases to 200 kPa, localized stress concentrations emerge, and the deepening color tones indicate that biochar particles begin to bear the primary load, initiating force chain development. At 800 kPa, high-stress regions expand and become more distinct, accompanied by potential biochar particle fracture, which redistributes internal stresses. At the highest loading stage (3200 kPa), most regions exhibit dark coloration, indicating elevated stress levels. At this stage, extensive biochar fragmentation disrupts previously continuous force chains, necessitating reconfiguration of the internal stress network.

**Figure 13:**
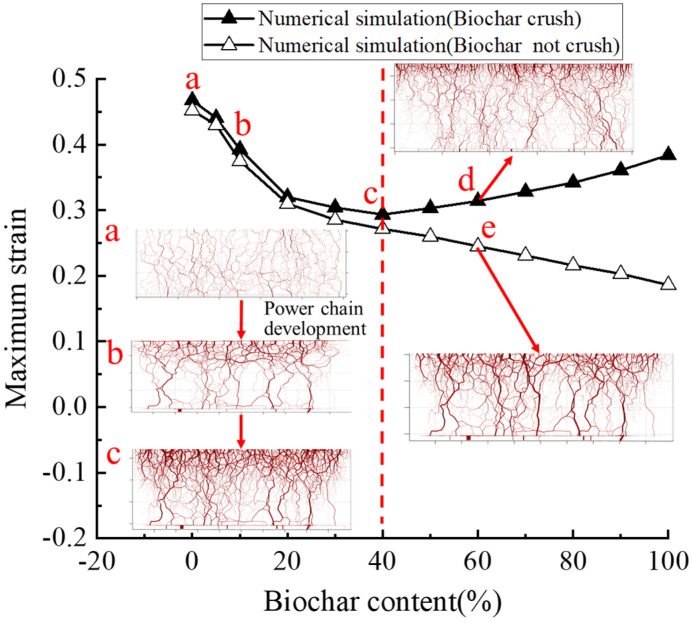
Internal stresses in mixed soil samples with different biochar content.

Figure 13 compares internal stress distributions in mixtures with varying biochar content. When biochar content is ≤40%, particle fragmentation is minimal, and the effect on soil compressibility is limited. Force chain diagrams for representative states (points a, b, and c) reveal that at low biochar content, soil particles primarily form the structural skeleton, with force chains appearing relatively fine, thick, and evenly distributed. As biochar content increases, contact between biochar particles becomes more frequent, with soil particles filling the voids between them. Biochar gradually transitions into a load-bearing skeletal component, increasing the number and strength of primary force chains and enhancing the mixture’s capacity to resist external loads. However, when biochar content exceeds 40%, the effect of particle crushing on compressibility becomes pronounced. Numerical simulations indicate that, under these conditions, the force chains weaken and become more uniformly distributed when biochar undergoes crushing, thereby reducing the mixture’s ability to inhibit deformation.

**Figure 14:**
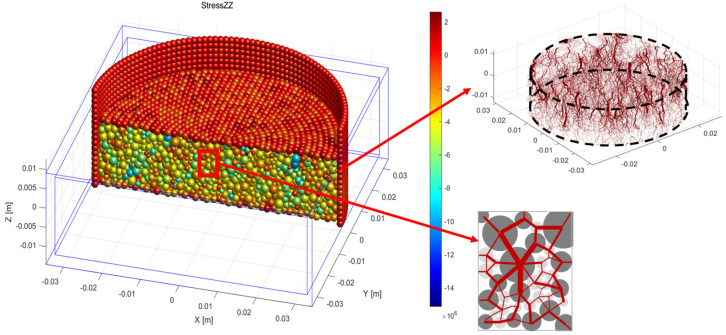
Three-dimensional force chain distribution and particle connections in the mixed soil sample.

Figure 14 illustrates the three-dimensional distribution of force chains in the mixed soil sample. Biochar particles demonstrate more extensive interactions with surrounding particles and exhibit superior load transfer capacity compared to soil particles. This behavior indicates that biochar particles predominantly bear the applied stresses, while soil particles primarily function to stabilize the overall skeleton. This load-sharing dynamic effectively suppresses compression and deformation in the biochar–soil mixtures.

### 4.2. Effect of Biochar Particle Crushing on Soil Compression Properties

**Figure 15:**
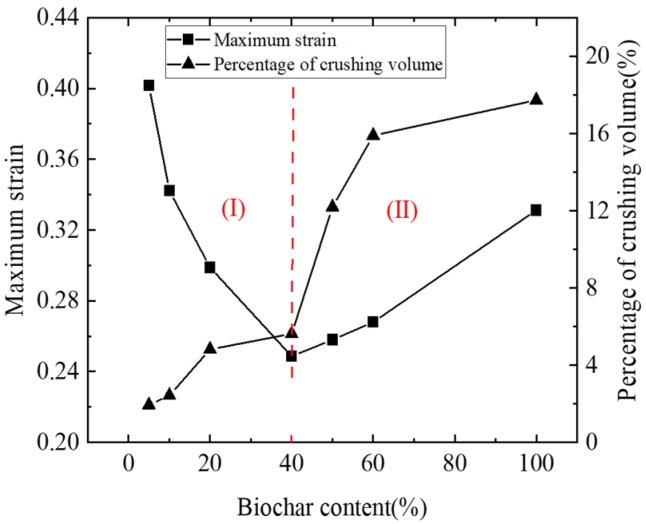
Relationship between biochar crushing rate and maximum strain of mixed soil samples. Stage I indicates reduced compressibility, while Stage II represents increased compressibility caused by biochar crushing.

Figure 15 depicts the relationship between particle breakage rate and maximum strain in biochar–soil mixtures with varying biochar contents. Overall, the crushing rate increases with biochar content. In Stage I, the compressibility of the mixture decreases as biochar content rises. This trend can be attributed to two factors: (i) the lower density of biochar reduces the initial sample volume and increases bulk density, and (ii) the relatively higher hardness of biochar particles compared to soil particles enhances the mixture’s resistance to deformation. Although minor biochar fragmentation occurs in this stage, its effect on compressibility is negligible, and the dominant influence is the inhibition of soil deformation.

Particle fragmentation is closely linked to force chain distribution, with breakage occurring more readily within strong force chains than in weak ones. Such breakage reduces force chain strength, but as loading continues, new force chains form, and the system approaches a new equilibrium state [59]. In Stage II, further increases in biochar content result in a higher crushing rate, altering the particle-size distribution of the mixture. This change reduces the average coordination number, weakens the skeletal load-bearing capacity, and markedly increases compressibility. Consequently, soil strain rises, producing the upward trend observed in this stage. These findings are consistent with Reddy et al. (2015) [9], who reported that compressibility increases markedly when biochar content reaches 100%.

In summary, the incorporation of an optimal amount of biochar can effectively suppress soil compressibility. However, beyond a critical threshold, excessive biochar undergoes extensive fragmentation under consolidation stress, diminishing its capacity to improve soil mechanical performance.

### 4.3. Variation in the Biochar Fragmentation Rate Under Stepwise Loading

**Figure 16:**
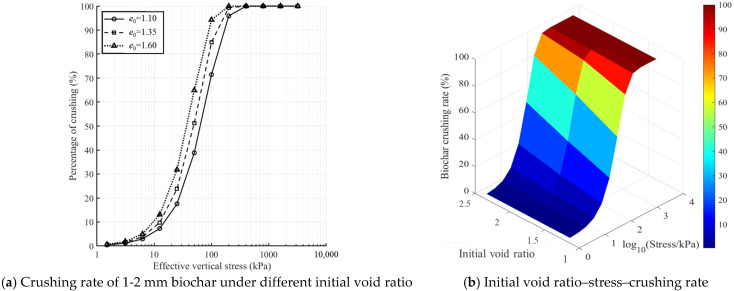
Changes in the crushing rate of biochar.

Figure 16 illustrates the variation in biochar crushing rate. As shown in Figure 16a, the crushing rate of 1–2 mm biochar under different initial void ratios exhibits distinct stress-dependent zones. In the low-stress zone (<100 kPa), the slope of the crushing rate–stress curve is small, reflecting the initial compaction stage. In the medium-stress zone (100–800 kPa), the slope increases sharply, indicating progressive damage to the particle skeleton. In the high-stress zone (>800 kPa), the curve flattens, suggesting that particle breakage approaches saturation. Under low-stress conditions, void ratio primarily influences crushing by modifying the contact state between particles. In contrast, at high stress, internal defects within the particles become the dominant factor controlling breakage, thereby reducing the relative influence of void ratio [45,59].

As shown in Figure 16b, the three-dimensional response surface of void ratio, stress, and crushing rate exhibits a consistent upward trend along the stress axis. The contour lines become denser in the high-stress region, further confirming that the effect of void ratio diminishes as stress increases.

From a practical perspective, when applying biochar for soil improvement under high-void-ratio conditions, graded loading (≤100 kPa per stage) is recommended. This incremental approach allows internal stress and deformation to accumulate gradually, providing sufficient time for particle rearrangement and structural adjustment. Such a loading strategy minimizes localized damage and large-scale biochar crushing caused by abrupt stress increases, thereby ensuring more effective and durable improvement of soil properties.

## 5. Conclusions

To investigate the influence of biochar on the compressive behavior of soil, this study employed the discrete element software MatDEM to construct a three-dimensional discrete element model incorporating biochar particle crushing. The key findings are as follows:

(1) Appropriate addition of biochar can reduce soil compressibility by enhancing the stability of the soil skeleton. However, the numerical simulations conducted in this study indicate that when the biochar content exceeds approximately 40%, particle crushing becomes more pronounced, which in turn increases compressibility.
(2) Force chain analysis demonstrates that biochar particles, owing to their larger contact area and higher stiffness relative to mineral soil particles, establish stable principal stress-transfer pathways during the initial loading stage. These pathways provide both skeletal support and stress dispersion, delaying structural failure. The robust force chains formed by biochar are the primary mechanism responsible for enhancing compressive strength, while the native soil particles predominantly contribute to maintaining the mixture’s overall stability.
(3) A higher initial void ratio reduces interparticle constraints, loosens the soil structure, and facilitates microcrack propagation. Larger biochar particles are more susceptible to macro-fractures under stress concentration in soils with fewer contact points. These conditions increase fines production, reorganize the pore structure, elevate particle breakage rates, refine particle size distribution, and reduce coordination numbers, thereby diminishing the load-bearing capacity of the soil skeleton and exacerbating compressive deformation.
(4) Biochar crushing proceeds through three stress-dependent stages: (i) initial compaction (<100 kPa), (ii) rapid skeletal damage (100–800 kPa), and (iii) crushing saturation (>800 kPa). At low stress, void ratio is the primary factor influencing crushing, whereas at high stress, internal defects within the biochar particles dominate. To mitigate damage and enhance soil stability, graded loading with stress increments of ≤100 kPa per stage is recommended.

Current research still has some limitations. Future studies could further conduct comparative investigations under different soil conditions and deeply explore the mechanism of biochar’s action in complex operating conditions by incorporating long-term complex environmental effects.

## Figures and Tables

**Table 1:** Parameters of soil and biochar used in the simulation [7,34,35].

Parameters	Value	
	Soil	Biochar
Young’s modulus (*E*/GPa)	0.04	10
Poisson’s ratio (*ν*)	0.25	0.3
Tensile strength (*T*_u_/MPa)	0.01	5
Compression strength (*C*_u_/MPa)	0.1	9
Coefficient of intrinsic friction (*μ*_i_)	0.6	0.4
Density (*ρ*/kg/m^3^)	1800	470
Particle size (mm)	0~0.005	0.5~1, 1~2, 2~3

## Data Availability

The original contributions presented in this study are included in the article. Further inquiries can be directed to the corresponding author.

## Abbreviations

The following abbreviations are used in this manuscript:

CT	Computed Tomography
CEC	Cation Exchange Capacity
MRPE	Mean Relative Percentage Error
3D	Three-Dimensional
